# Safety analysis of holmium-166 microsphere scout dose imaging during radioembolisation work-up: A cohort study

**DOI:** 10.1007/s00330-017-4998-2

**Published:** 2017-08-07

**Authors:** Arthur J. A. T. Braat, Jip F. Prince, Rob van Rooij, Rutger C. G. Bruijnen, Maurice A. A. J. van den Bosch, Marnix G. E. H. Lam

**Affiliations:** Department of Radiology and Nuclear Medicine, University Medical Center Utrecht, Heidelberglaan 100, Huispostnummer E01.132, 3584 CX Utrecht, The Netherlands

**Keywords:** Radioembolisation, SIRT, Holmium, Embolisation, therapeutic, Technetium Tc-99m Aggregated Albumin

## Abstract

**Objective:**

Radioembolisation is generally preceded by a scout dose of technetium-99m-macroaggregated albumin to estimate extrahepatic shunting of activity. Holmium-166 microspheres can be used as a scout dose (±250 MBq) and as a therapeutic dose. The general toxicity of a holmium-166 scout dose (^166^Ho-SD) and safety concerns of an accidental extrahepatic deposition of ^166^Ho-SD were investigated.

**Methods:**

All patients who received a ^166^Ho-SD in our institute were reviewed for general toxicity and extrahepatic depositions. The absorbed dose in extrahepatic tissue was calculated on SPECT/CT and correlated to clinical toxicities.

**Results:**

In total, 82 patients were included. No relevant clinical toxicity occurred. Six patients had an extrahepatic deposition of ^166^Ho-SD (median administered activity 270 MBq). The extrahepatic depositions (median activity 3.7 MBq) were located in the duodenum (3x), gastric fundus, falciform ligament and the lesser curvature of the stomach, and were deposited in a median volume of 15.3 ml, which resulted in an estimated median absorbed dose of 3.6 Gy (range 0.3–13.8 Gy). No adverse events related to the extrahepatic deposition of the ^166^Ho-SD occurred after a median follow-up of 4 months (range 1–12 months).

**Conclusion:**

These results support the safety of 250 MBq ^166^Ho-SD in a clinical setting.

***Key Points*:**

• *A holmium-166 scout dose is safe in a clinical setting.*

• *Holmium-166 scout dose is a safe alternative for* ^*99m*^ *Tc-MAA for radioembolisation work-up.*

• *Holmium-166 scout dose potentially has several benefits over* ^*99m*^ *Tc-MAA for radioembolisation work-up.*

## Introduction

Before yttrium-90 (^90^Y) radioembolisation (RE) is performed, a scout dose is used to predict intra- and extrahepatic distribution of activity and check for potential contraindications (i.e. excessive lung shunt and extrahepatic depositions). Technetium-99m macro aggregated albumin (^99m^Tc-MAA) is commonly used; however, its predictive value has been discussed in the literature [1, 2]. In patients treated with holmium-166 (^166^Ho) microspheres a scout dose using 250 MBq ^166^Ho is used as an alternative, which is superior in calculating the lung shunt fraction compared to ^99m^Tc-MAA [1]. This may be due to the fact that identical ^166^Ho microspheres are used for the scout dose procedure and the RE treatment.

The beta- and gamma-emitting properties of ^166^Ho (respectively E_βmax_ = 1.85 MeV and E_γ_ = 81 keV) may theoretically raise concerns about the safety of using ^166^Ho microspheres as a scout dose. An earlier study concluded that ^166^Ho microspheres can safely replace ^99m^Tc-MAA in the majority of cases [3]. However, these data were based on ^99m^Tc-MAA data of extrahepatic depositions, theoretically translated to ^166^Ho microspheres; if these extrahepatic ^99m^Tc-MAA depositions had been ^166^Ho microspheres, only 5.9% of patients would have excessive absorbed doses in extrahepatic tissues [3]. This toxicity assessment was performed because of a lack of events after ^166^Ho microsphere scout dose procedures.

Since then, a ^166^Ho microsphere scout dose (^166^Ho scout dose) has been used in several clinical trials. Several events in the use of an extrahepatic ^166^Ho scout dose were observed that warrant a re-evaluation of the previous theoretically based safety assumptions. To assess the safety of the ^166^Ho scout dose in clinical practice, the general toxicity of a ^166^Ho scout dose was studied. Additionally, the absorbed dose in extrahepatic tissue in all patients with an extrahepatic ^166^Ho scout dose deposition was calculated and clinical record forms for potential complications due to these extrahepatic depositions were reviewed.

## Methods and materials

### Patient population

All patients who had been treated with ^166^Ho microspheres since the start of its clinical use were included, i.e. from November 2009 till January 2016. All patients included in this study participated in a prospective trial with ^166^Ho microspheres (Table 1 [4–7]) and written informed consent was obtained for all patients at study inclusion. All data were gathered prospectively and all studies were approved by the institution’s Ethics Committee prior to patient inclusion. Results of 15 patients treated with ^166^Ho radioembolisation in the HEPAR trial have been published previously [4]. This earlier article dealt with development of the ^166^Ho microspheres as a therapeutic agent, whereas this current study provides additional information solely on the toxicity of the ^166^Ho scout dose of those patients. The scout dose with ^166^Ho microspheres was aimed at 250 MBq in all study protocols and administered intra-arterially. The 250 MBq was divided amongst the injection positions according to the targeted liver volume. All patients received the scout dose administration in the morning prior to the therapeutic ^166^Ho dose administration in the afternoon on the same day. In patients with an extrahepatic deposition, additional volume, activity and dose quantification on imaging studies were performed and discussed separately (details in the next sections). Clinical record forms were evaluated for any adverse events during or after the ^166^Ho scout dose procedure and prior to the radioembolisation treatment with ^166^Ho microspheres. They were scored according to the Common Toxicity Criteria for Adverse Events (CTCAE) version 4.03. Angiography procedures were performed by experienced interventional radiologists (>3 years’ experience) and SPECT/CT readings by experienced nuclear medicine physicians (>3 years’ experience).

**Table 1:** Trials with ^166^Ho-microspheres

Trial	N	Follow-up period	^99m^Tc-MAA*	Description
HEPAR [4]	15	12 months	Yes	Phase 1 trial
HEPAR 2 [5]	42	12 months	Yes	Phase 2 trial
HEPAR PLUS [6]	13	12 months	No	Additional ^166^Ho radioembolisation after PRRT in NET
SIM [7]	11	3 months	No	Surefire Infusion System versus standard microcatheter use during ^166^Ho radioembolisation in CRLM

*PRRT* peptide receptor radionuclide therapy, *NET* neuroendocrine tumours, *CRLM* colorectal liver metastasis

*Use of ^99m^Tc-MAA prior to the ^166^Ho scout dose and actual treatment

### Imaging and reconstruction

Our phantom and all patients were scanned on a Symbia T16 SPECT/CT scanner (Siemens Healthcare, Erlangen, Germany) within 1 h of the injection of the ^166^Ho scout dose. Similar to ^99m^Tc-MAA, lung shunt fraction (LSF) was determined by drawing regions-of-interest (ROIs) of the lungs and the liver on anterior and posterior planar imaging of the thorax and abdomen. By calculating the fraction of the total activity using the geometric mean, resulting in the following equation:

$$ LSF=\frac{\sqrt{\left( lung{s}_{anterior}* lung{s}_{posterior}\right)}}{\sqrt{\left( lung{s}_{anterior}* lung{s}_{posterior}\right)}+\sqrt{\left( live{r}_{anterior}* live{r}_{posterior}\right)}} $$

SPECT data of the liver were acquired using a medium-energy general purpose collimator, on a 128 × 128 matrix (pixel size, 4.8 × 4.8 mm) with 120 angles (20 s per projection) over a non-circular 360° orbit and photonpeak energy window centred around 81 keV with a width of 15%. Low-dose CT data (110 kVp, 40 mAs, adaptive dose modulation with Siemens CARE Dose 4D) were acquired and reconstructed to a voxel size of 1.27 × 1.27 × 5 mm using a smoothing kernel (B08s; Siemens Healthcare). After a CT-derived attenuation map was created (Syngo MI Applications; Siemens Healthcare), quantitative SPECT images were reconstructed with ten iterations, with eight subsets using the Utrecht Monte Carlo System (UMCS), an in-house-developed Monte Carlo simulator, incorporating Monte Carlo-based scatter correction, attenuation correction, and modelling of photon interaction with the collimator and detector [8–10].

### Phantom equipment

To estimate the accuracy of our measurement, a National Electrical Manufacturers Association (NEMA) NU2 image quality (IQ) phantom was used. This contains six spheres of sizes varying between 0.5 and 26.5 ml suspended in a water-filled background compartment of 9.7 L. All spheres were filled with a ^166^Ho acidic solution of known activity concentration and scanned identically to the protocol used for the ^166^Ho scout dose SPECT/CT, as described previously [8].

### Activity and volume analysis of a deposition

Activity and volume estimation was carried out similarly to the earlier study using additional in-house-developed software (Volumetool) [3, 11]. Manual delineation of the extrahepatic deposition was performed by taking a large enough margin around the extrahepatic deposition to include all displaced counts due to breathing, patient motion and partial volume effects (excluding intrahepatic activity). The extrahepatic activity was estimated by summation of all voxels (in units of Bq), without the use of a threshold, within the manually delineated extrahepatic deposition, preventing underestimation of the extrahepatic activity (and thus of absorbed dose).

The threshold for volume delineation was determined in our phantom study and was defined as a percentage of the maximum voxel value. The threshold was applied to the same manual delineation in our case series to determine the extrahepatic deposition volume. A threshold was chosen to approximate or underestimate the volume (but not overestimate). This resulted in an overestimation of the extrahepatic tissue absorbed dose, which should decrease the possibility of a type II error: failure to reject the null hypothesis “Use of ^166^Ho scout dose is safe”.

### Dose calculation

The following formula was used to calculate the absorbed dose in the extrahepatic tissue:

$$ D(Gy)=15.87\left(\frac{mJ}{MBq}\right)*\frac{Extrahepatic\  activity\  of\ {}{}^{166}Ho\ (MBq)}{Extrahepatic\  volume\  of\ {}{}^{166}Ho\left(c{m}^3\right)*1.06\left(\frac{g}{c{m}^3}\right)} $$

where 15.87 mJ/MBq is the total energy absorbed in tissue from the beta decay of 1 MBq of ^166^Ho assuming soft tissue density of 1.06 g/cm^3^ [12]. The mean penetration of the beta emission of ^166^Ho (2.5 mm) is small, so all energy was assumed to be absorbed within the extrahepatic deposition [3]. The beta radiation accounts for 96% of the emitted energy (=15.87 mJ/MBq), the other 4% of the energy is for the most part emitted by high-energy (>1 MeV) gammas. Because of the large penetration distance of these gamma's, and the inverse square law, the absorbed radiation dose to surrounding tissues, due to the high-energy gamma’s, is negligible.

## Results

A total of 90 patients were included in the trials. After the initial ^99m^Tc-MAA procedure in the HEPAR and HEPAR-2 trial, eight patients were excluded. Five patients were excluded based on earlier ^99m^Tc-MAA findings (excessive lung shunt or extrahepatic deposition) and three due to technical reasons (dissection resulting in a permanent stenosis or new collaterals). A total of 82 patients with moderate to extensive bilobar disease received a ^166^Ho scout dose at our institute (Table 2). A mean scout dose of 244 MBq was administered (median 251 MBq; range 103–313 MBq). Six patients (7.9%) had an extrahepatic deposition, which will be discussed in detail in the next section. Table 3 provides all the adverse events after the scout dose administration (prior to therapeutic dose administration) and adverse events related to the pre-treatment angiography procedure. No adverse events that were possibly, probably or definitely related to the ^166^Ho scout dose occurred.

**Table 2:** Baseline characteristics of patients receiving a ^166^Ho scout dose

Age (years)	
Mean	62.2
Standard deviation	10.5
Range	38 – 88
Primary tumour (n)	
Colorectal carcinoma	43
Neuroendocrine tumour	16
Orbital melanoma	8
Cholangiocarcinoma	5
Breast cancer	5
Pancreatic adenocarcinoma	2
Appendix carcinoma	1
Gastric cancer	1
Thymoma	1
Scout dose	
Mean prescribed dose (MBq)	265
Range	105– 326
Mean net administered dose (MBq)	242
Range	103 – 313
Mean lung shunt fraction (%)	13.2
Range	1.1 – 24.9
Treatment planning	
Whole liver, one session	80
Whole liver, sequentially	2

**Table 3:** Adverse events surrounding scout dose administration

Adverse event	CTCAE grade*	N	%
Back pain^†^	1	5	6.3
	2	3	3.8
Abdominal pain^‡^	1	2	2.5
Dissection		2	2.5
Stenosis right hepatic artery		1	1.3
Allergic reaction to iodine contrast		1	1.3

*Common Terminology Criteria for Adverse Events version 4.03

^†^Back pain related to angiography suite table or SPECT/CT table

^‡^Abdominal pain occurred after coiling of a phrenic artery, before ^166^Ho scout dose administration

### Extrahepatic depositions ^166^Ho scout dose

**Fig. 1:**
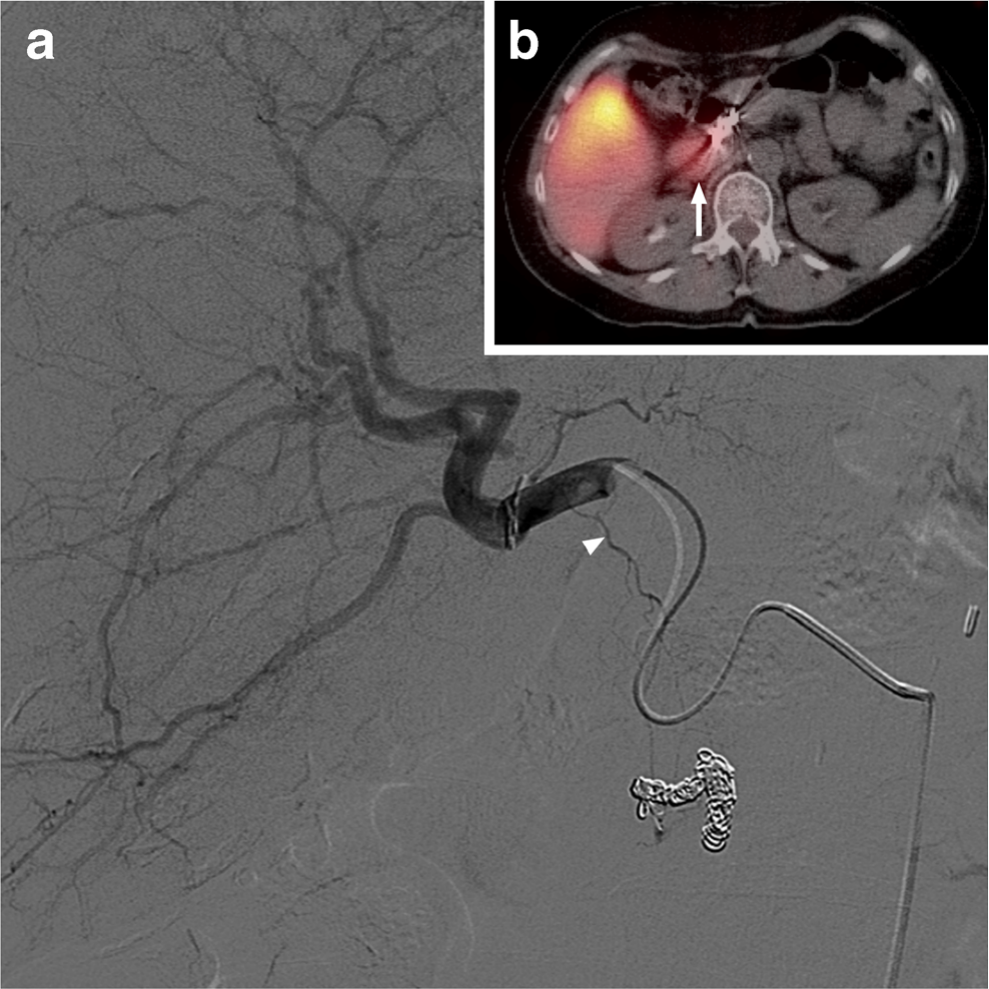
A 63-year-old female with an intrahepatic cholangiocarcinoma. (**a**) Digital subtraction angiography (DSA) image with injection position in right hepatic artery. (**b**) ^166^Ho scout dose SPECT/CT with duodenal extrahepatic deposition (arrow). On DSA, posterior superior pancreatico-duodenal artery was the culprit vessel (arrow), which in 15% of cases originates from the common hepatic artery or main hepatic artery [13]. However, it can also arise from the right hepatic artery [14], as illustrated here

**Fig. 2:**
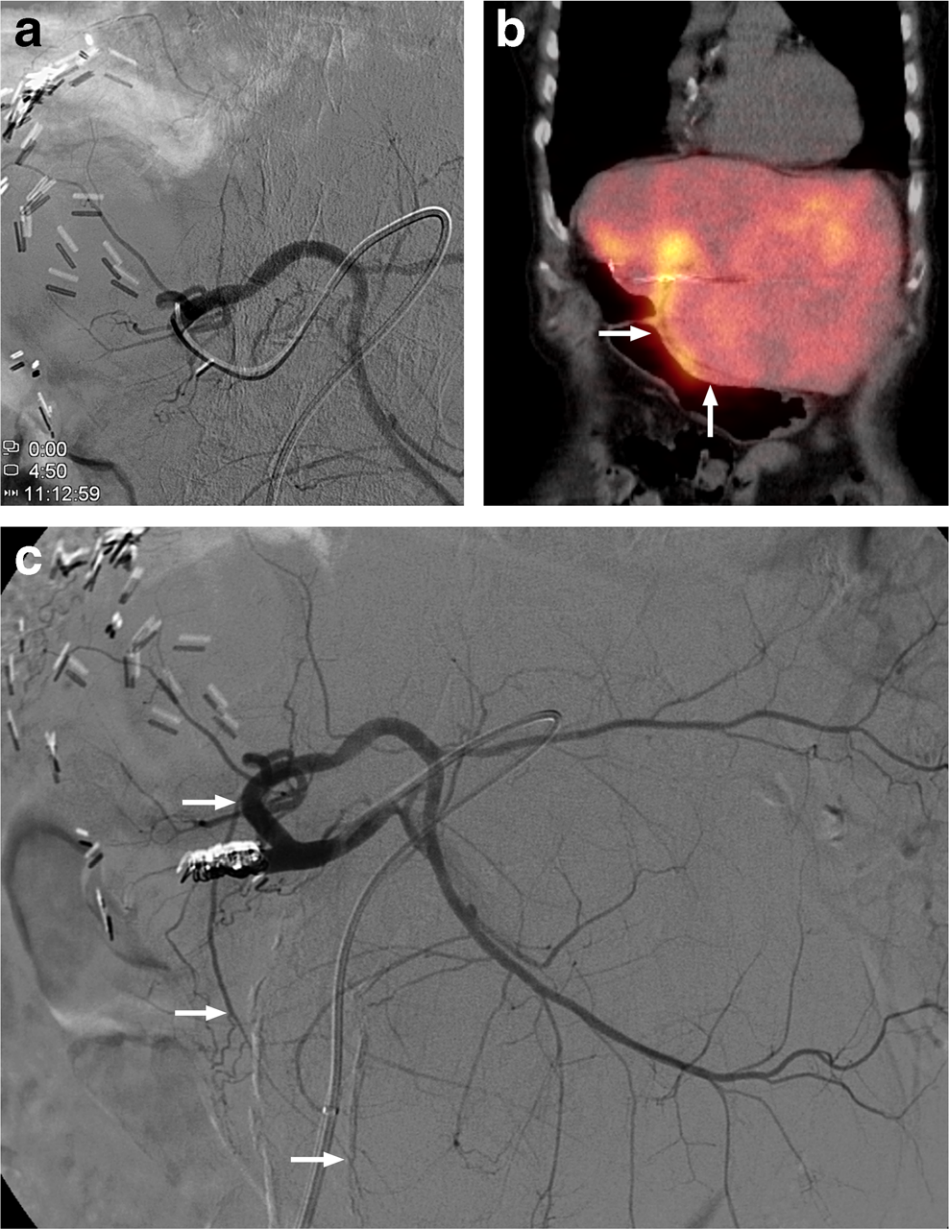
An 80-year-old female with colorectal cancer liver metastases was initially treated with a resection of her sigmoid carcinoma and simultaneous right hemihepatectomy. (**a**) Digital subtraction angiography (DSA) with injection position during ^166^Ho scout dose pre-treatment angiography. (**b**) SPECT/CT after administration of ^166^Ho scout dose with large extrahepatic deposition in lesser curvature of the stomach (arrows). (**c**) DSA showing the right gastric artery arising from the right hepatic artery as culprit vessel (arrows)

**Fig. 3:**
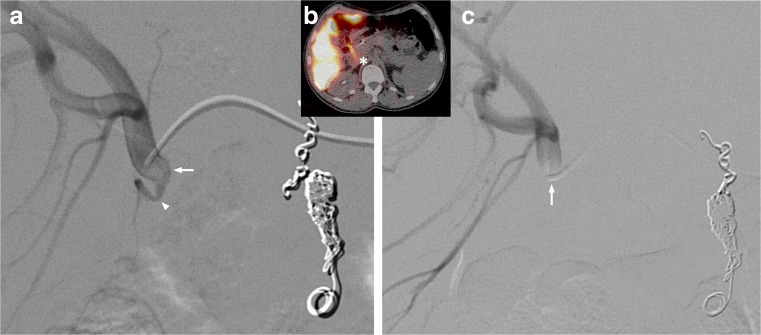
A 63-year-old male with colorectal cancer liver metastases. (**a**) Digital subtraction angiogram (DSA) of injection position of ^166^Ho scout dose procedure. (**b**) ^166^Ho scout dose SPECT/CT with extrahepatic deposition in duodenum (star). (**c**) DSA of injection position of ^166^Ho therapy. Note the difference in positioning of microcatheter (arrows). During ^166^Ho scout dose procedure the microcatheter pointed downwards, instead of horizontally (arrows). On the same DSA of ^166^Ho scout dose procedure the culprit vessel, supraduodenal artery, can be identified (arrowhead)

**Fig. 4:**
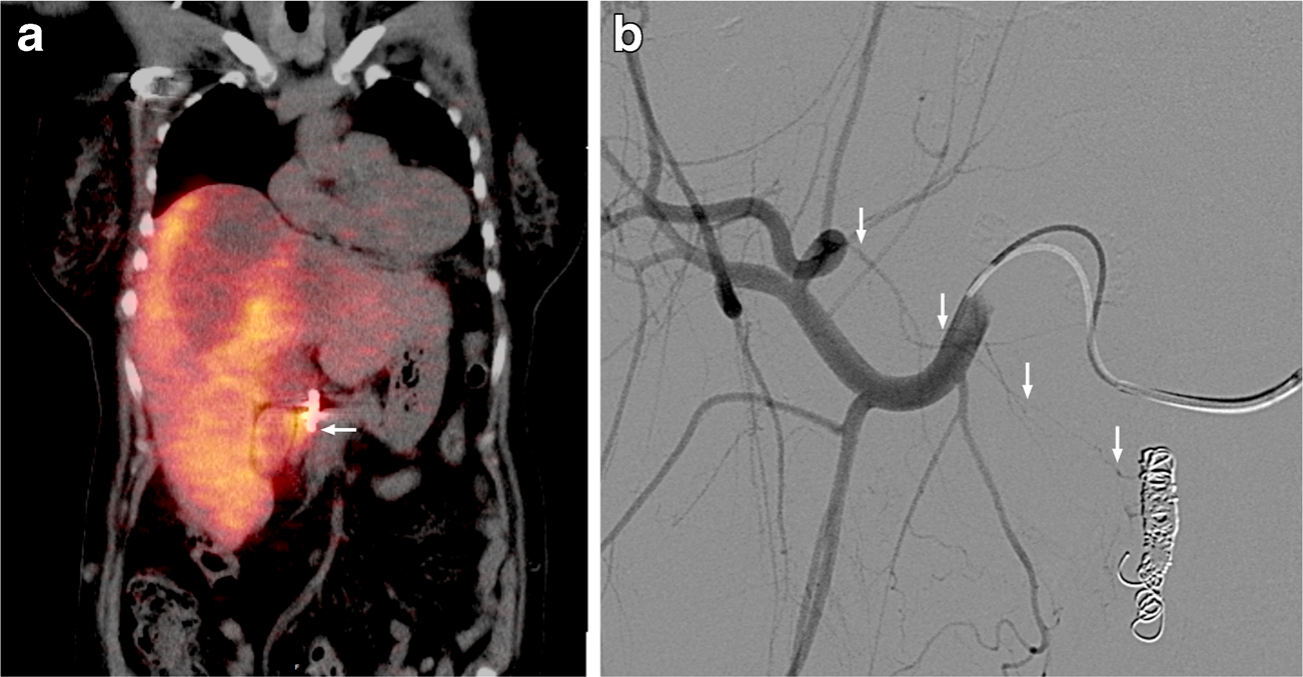
A 62-year-old female with liver metastases of a pancreatic adenocarcinoma. (**a**) SPECT/CT after administration of ^166^Ho scout dose. Extrahepatic deposition in the duodenum (arrow). (**b**) Digital subtraction angiography shows flow redistribution in intrahepatic collateral (arrows), directly following coil embolization of gastroduodenal artery. Development of new hepatico-enteric collaterals after previous coil embolization has been described before [15]

**Fig. 5:**
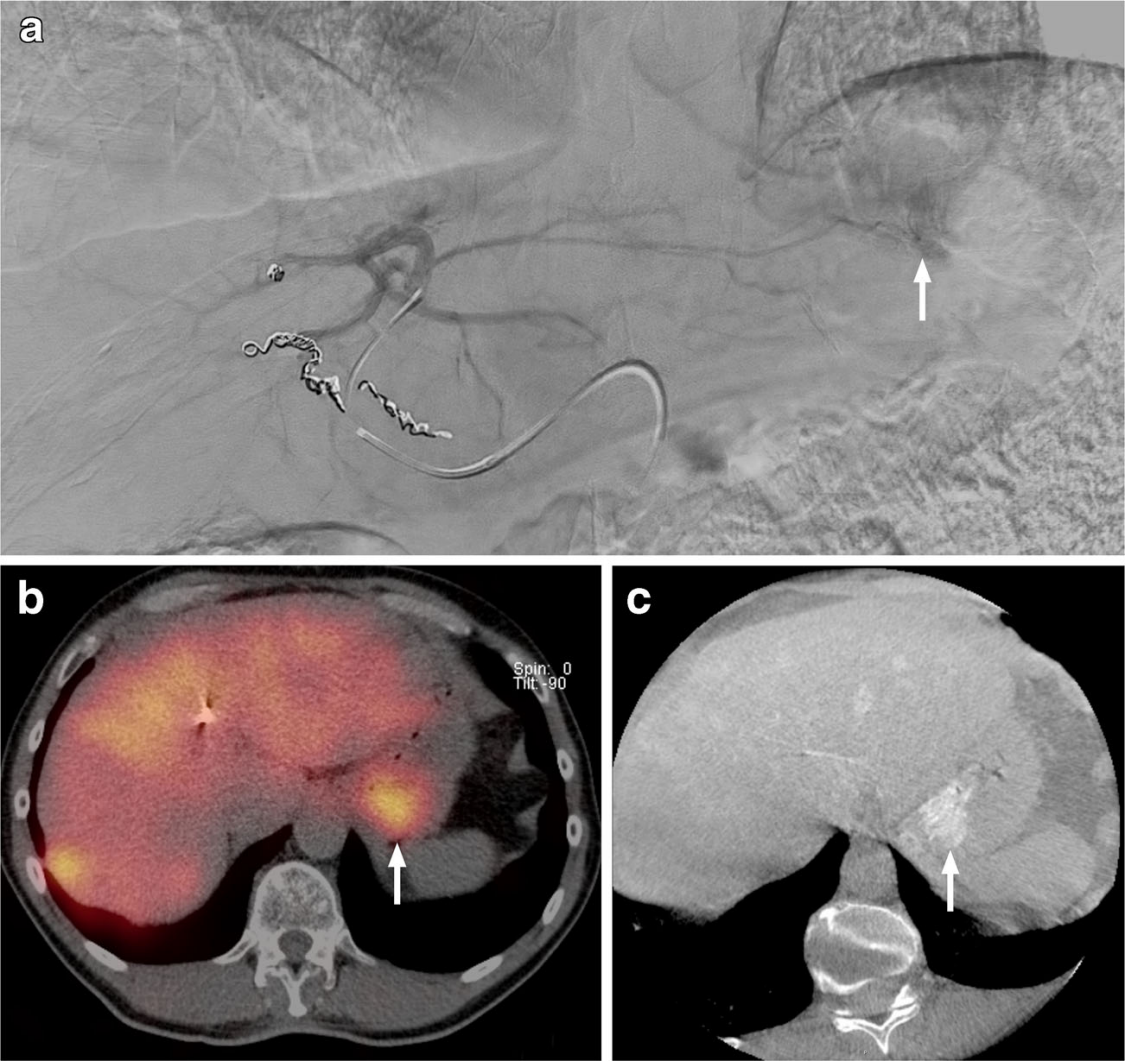
A 56-year-old male with a primary rectal neuroendocrine tumour, liver and bone metastases. (**a**) Digital subtraction angiography of ^166^Ho scout dose procedure. (**b**) Corresponding ^166^Ho scout dose SPECT/CT. (**c**) Corresponding cone beam CT. All images show extrahepatic deposition and contrast blush in gastric fundus (arrows). After coiling of accessory left gastric artery, extrahepatic deposition on SPECT/CT and contrast blush on cone beam CT disappeared (images not shown). Accessory left gastric artery originated distally from left hepatic artery, running through the ligamentum venosum towards the gastric fundus [16]

**Fig. 6:**
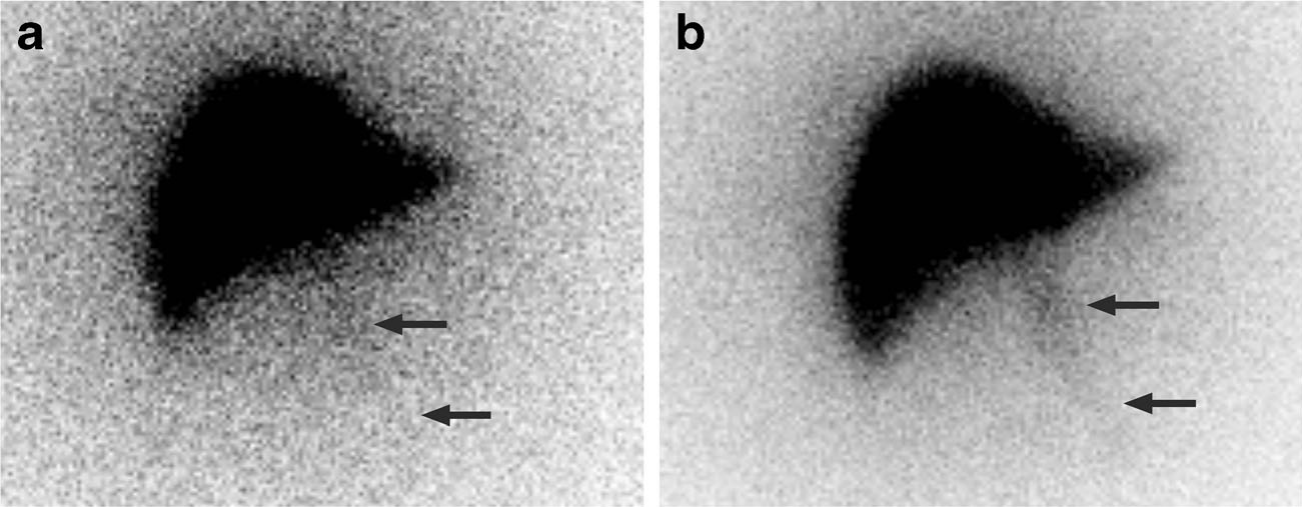
A 60-year-old male with colorectal liver metastases. (**a**) Planar ^166^Ho image of scout dose, depicting a faint extrahepatic deposition in falciform ligament. (**b**) Post-treatment planar ^166^Ho image, depicting similar extrahepatic deposition in falciform ligament (SPECT/CT images not shown)

Six patients had an extrahepatic deposition of the ^166^Ho scout dose (Figs. 1, 2, 3, 4, 5 and 6): one HEPAR, three HEPAR 2, one HEPAR PLUS and one SIM candidate [4–7]. Baseline characteristics can be found in Table 4. Median LSF (of these patients) was 13.3% (range 9.4–17.6). Median follow-up was 4 months (range: 1–12 months).

**Fig. 7:**
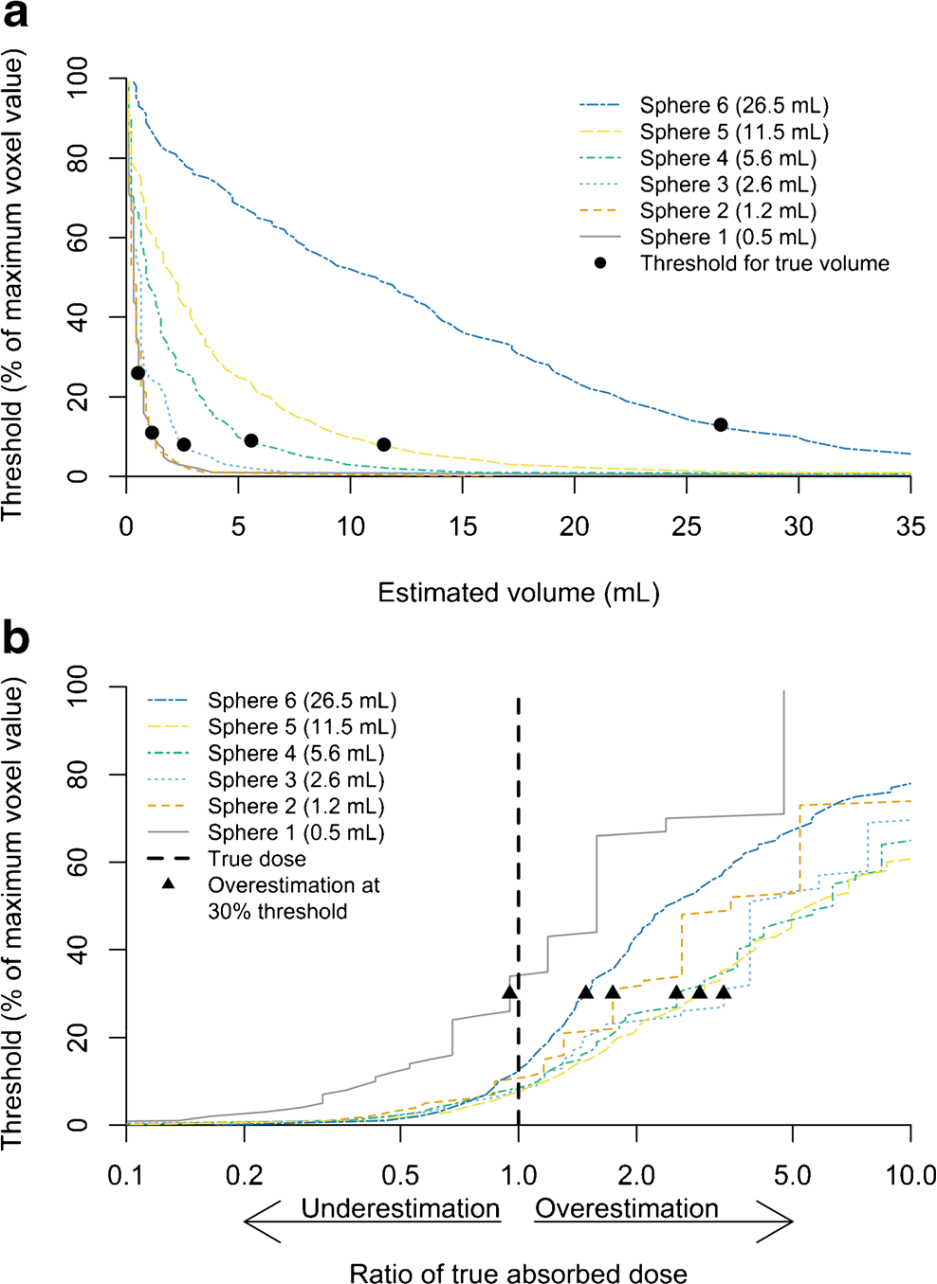
(**a**) Graph featuring results of threshold-based volume estimation of six ^166^Ho-filled spheres in NEMA NU-2 Image Quality phantom (0.5–26.5 ml). (**b**) Threshold-estimated absorbed doses relative to their true value in phantom spheres, using 30% threshold and known ^166^Ho acidic solution concentration. Underestimation of affected tissue volume in vivo will occur, subsequently leading to overestimation of absorbed tissue dose (up to four times in sphere 3 of 2.6 ml). A slight underestimation of the absorbed dose will only occur in small-volume extrahepatic depositions (<1 ml)

**Table 4:** Patients with an extrahepatic deposition of a ^166^Ho scout dose

Patient	Figure	Age	Primary tumour	Location	Culprit vessel	Treated	Volume* (ml)	Activity (MBq)	Estimated Dose (Gy)
1	2	63	Intrahepatic cholangiocarcinoma	Duodenum	Posterior superior pancreatico-duodenal artery	Yes	13.9	1.3	1.4
2	3	80	Sigmoid carcinoma	Lesser curvature of the stomach	Right gastric artery	No	35.5	33.1	13.8
3	4	63	Colon carcinoma	Duodenum	Supraduodenal artery	Yes	15.4	2.3	2.2
4	5	62	Pancreas adenocarcinoma	Duodenum	Intrahepatic collateral	No	15.3	3.7	3.6
5	6	56	Neuroendocrine tumour	Gastric fundus	Accessory left gastric artery	Yes	9.6	7.4	11.7
6	7	60	Colon carcinoma	Falciform ligament	Falciform artery	Yes	9.2	0.2	0.3

*Extrahepatic deposition volume was based on the 30% threshold of the phantom study (see Fig. 1)

Patients 2 and 4, respectively depicted in Figs. 2 and 4, were excluded from treatment, as the culprit vessels remained unidentified, so treatment was deemed unsafe. After focused reviewing of the old DSA images several years later, probable culprit vessels were identified. In patient 2 the right gastric artery was the probable culprit vessel, as its origin was exactly at the tip of the microcatheter during the ^166^Ho scout dose injection (Fig. 2). In patient 4 an intrahepatic collateral was the culprit vessel, which fed the region of the coil-embolised gastroduodenal artery (Fig. 4).

### Dosimetry and follow-up

#### Results of the phantom study

A threshold of 30% of the maximum voxel value was chosen based on our phantom study because it provided an underestimation of the volume in all spheres (Fig. 7a). Using this threshold, the absorbed dose in only the smallest sphere (0.5 ml) was underestimated (Fig. 7b; due to simultaneous underestimation of the activity). This was deemed irrelevant from a clinical point of view, because the earlier published study with ^99m^Tc-MAA data had no depositions smaller than 1 ml in a larger patient population [3].

#### Patient data

Based on the ^166^Ho scout dose SPECT/CT, the absorbed dose on extrahepatic tissues by the scout dose was assessed (Table 4). Calculations in these patients showed a median extrahepatic deposition volume of 15.3 ml (range 9.2–35.5 ml) and median absorbed dose of 3.6 Gy (range 0.3–13.8 Gy). The maximum absorbed dose to extrahepatic tissues was 13.8 Gy. The method used conservatively underestimated the volume of the deposition to reduce the risk of underestimating the absorbed dose. Although extrahepatic depositions of ^166^Ho occurred, the resulting absorbed doses were estimated to be at most 14 Gy.

The extrahepatic deposition in patient 1, depicted in Fig. 1, was also seen on the earlier ^99m^Tc-MAA SPECT/CT and the culprit vessel was unidentified at the time of treatment (it was only identified after focused retrospective reviewing). Patient 1 was treated at that time, because she had no other therapeutic options and had very aggressive disease. Within several days, the patient developed abdominal pain (maximum CTCAE grade 4) and the post-treatment SPECT/CT showed the same extrahepatic deposition in the duodenum. Using the same quantitative SPECT-reconstruction method for the post-treatment imaging, a radiation-absorbed dose in the duodenum of 134.5 Gy was calculated. Endoscopy revealed an inflamed duodenal wall, which fitted a radiation-induced (non-erosive) duodenitis. No severe complications (e.g. perforation) occurred. Her duodenitis recovered after 6 weeks and she passed away 4 months after treatment due to progressive disease.

The extrahepatic deposition in the falciform ligament in patient 6, depicted in Fig. 6, was deemed irrelevant and the patient was treated the same day without ice packing of the abdominal skin. No clinical complications occurred (e.g. radiation dermatitis) during follow-up. Using the same quantitative SPECT-reconstruction method for the post-treatment imaging, a radiation absorbed dose in the falciform ligament of 33.5 Gy was calculated. Four months after the treatment, the patient started a new chemotherapeutic regimen due to progressive disease and was lost to follow-up.

## Discussion

As shown in this study, no adverse clinical events occurred that were related to the use of a ^166^Ho scout dose. Common adverse events related to the angiography table or procedure were seen and are similar to those seen in ^99m^Tc-MAA-related procedures. In patients with extrahepatic depositions of the scout dose, a maximum of 14 Gy in extrahepatic tissues was calculated. Although the calculated 14 Gy was overestimated due to the overestimation of the deposited activity and underestimation of the extrahepatic deposition volume, this absorbed dose was far below the limit of 49 Gy as suggested by Kao et al [17, 18]. These clinical results of the ^166^Ho scout dose confirm the previously published safety assumptions based on theoretical evaluation using ^99m^Tc-MAA data [3]. However, the limit of 49 Gy suggested by Kao et al. is based on just two patients [17, 18]. Additionally, the absorbed dose in extrahepatic tissue is being discussed in the literature, as the limits could be even higher depending on the extrahepatic tissue type. In six porcine models, absorbed doses greater than 50 Gy (up to 92 Gy) in the gastric fundus showed mucosal haemorrhage or small (healed) superficial ulcers without a severe complication (e.g. perforation) [19]. Nonetheless, dose limitations for different tissue types need to be investigated further.

Based on the HEPAR and HEPAR 2 data, using a ^99m^Tc-MAA procedure prior to the ^166^Ho scout dose procedure, the use of a ^166^Ho scout dose alone for radioembolisation assessment was deemed safe. Along with the suspected limited extrahepatic tissue dose and its safety, both supported by this study, we have skipped the ^99m^Tc-MAA procedure in more recent studies with ^166^Ho microspheres, namely the SIM and HEPAR-PLUS study [6, 7].

Using ^166^Ho microspheres as a scout dose could benefit patients. The variation in intrahepatic distribution between the scout dose and treatment dose is expected to be minimal, due to the identical morphology of the microspheres. This is important for accurate intrahepatic dosimetry, especially when using the so-called ‘partition calculation method’ or voxel-based dosimetry, which is based on SPECT/CT of the scout dose distribution [20]. Currently, it is known that a large intrahepatic variability between ^99m^Tc-MAA and ^90^Y microspheres exists, probably influenced by many factors, including tumour type and burden, particle flow dynamics and catheter positioning, but also particle morphology [2, 20, 21]. Additionally, unlike ^99m^Tc-MAA and free pertechnetate, ^166^Ho does not freely circulate in vivo. No unwanted uptake of activity in the stomach wall, kidneys, thyroid and lungs occurs. Due to its identical particle morphology and the absence of freely circulating ^166^Ho, a^166^Ho scout dose is superior in lung shunt fraction calculation compared to ^99m^Tc-MAA [1]. As shown by Elschot et al. [1] with SPECT/CT data, ^99m^Tc-MAA may overestimate the lung shunt fraction up to 170% when compared to ^166^Ho microspheres. Yu et al. also showed a significant overestimation of the lung shunt fraction by ^99m^Tc-MAA using standard planar scintigraphy compared to SPECT/CT [22]. Considering their data, most patients with a lung shunt fraction of >20% on planar ^99m^Tc-MAA imaging are wrongfully refused for radioembolisation treatment, which can be overcome by the use of a ^166^Ho scout dose.

The use of ^166^Ho microsphere scout dose imaging is safe, could lead to more reliable pre-treatment imaging and subsequently to improved individualised treatment planning. Another benefit of holmium is its large magnetic susceptibility (typical for most lanthanides), which may enable MRI-based dosimetry [23] and MR-guided treatments [24] in the future. Once MRI-based dosimetry is adequately developed, the scout dose may eventually be replaced for the stable, non-radioactive ^165^Ho microspheres.

Nonetheless, the fabrication process of ^166^Ho-microspheres is more complicated and time-consuming than ^99m^Tc-MAA, which is widely available through using one of the several commercially available ^99m^Tc-MAA kits for in-house production. Currently, commercial availability of ^166^Ho-microspheres is limited, but is expected to improve in the coming years.

The main limitation of this study was the fact that the safety analysis was limited by the number of events. However, theoretical and clinical analysis concordantly showed an acceptable low risk of toxicity. An additional limitation was the use of a pre-treatment angiography procedure with ^99m^Tc-MAA prior to the ^166^Ho scout dose procedure in the HEPAR and HEPAR 2 trials, resulting in a selection bias as five patients were excluded based on ^99m^Tc-MAA findings and these five patients did not receive a ^166^Ho scout dose. Four patients were excluded based on a ^99m^Tc-MAA extrahepatic deposition and one patient was excluded based on a ^99m^Tc-MAA lung shunt of 26.5%. Additionally, microcatheter positioning was altered based on the ^99m^Tc-MAA-SPECT/CT in another patient, to prevent an unwanted gallbladder deposition.

The calculation of absorbed dose is prone to the same limitations as described in the earlier publication [3]. The 30% threshold was based on a homogenous activity distribution in the spheres of our phantom versus a more heterogeneous accumulation of activity in extrahepatic depositions. Additionally, the phantom study is an ideal situation without breathing artefacts or patient movements.

In none of the six patients with an extrahepatic deposition of ^166^Ho microspheres were cone beam CTs performed prior to injection of the scout dose. Without the use of cone beam CTs, up to 6.5% of the cases still have an extrahepatic deposition on the SPECT/CT, when DSA is negative [25].

The introduction of improved pre-treatment CT imaging and cone beam CT during the radioembolisation procedures has probably contributed to a decrease in the number (and probably also extent) of extrahepatic depositions [20]. The use of cone beam CT has improved the detection of potential culprit vessels during the angiography procedure. The use of a scout dose SPECT/CT is therefore debated in the literature [26–28]. Nonetheless, once both DSA and cone beam CT are negative, the use of ^166^Ho microspheres for the detection of extrahepatic depositions becomes even safer.

Additionally, the 81 keV gamma-emitting properties of ^166^Ho make dual-isotope imaging with ^99m^Tc compounds possible. We have developed a dual-isotope protocol using ^166^Ho scout dose and ^99m^Tc stannous phytate, respectively, for microsphere distribution and healthy liver parenchyma delineation [29]. Using this combination, dose-volume histograms of healthy liver parenchyma can be easily calculated and allows the physician to calculate the maximum, safe to use therapeutic dose in a patient. The main advantage is the quick insight in the dose to healthy liver parenchyma, which is the main limiting factor in all radioembolisation treatments.

## Conclusion

This study clinically supports the previously stated hypothesis that the use of ^166^Ho microspheres as a scout dose (250 MBq) prior to radioembolisation is a safe alternative for ^99m^Tc-MAA.
